# Exchange of amino acids in the H1-haemagglutinin to H3 residues is required for efficient influenza A virus replication and pathology in *Tmprss2* knock-out mice

**DOI:** 10.1099/jgv.0.001128

**Published:** 2018-08-06

**Authors:** Ruth L. O. Lambertz, Jan Pippel, Ingo Gerhauser, Heike Kollmus, Darisuren Anhlan, Eike R. Hrincius, Joern Krausze, Nora Kühn, Klaus Schughart

**Affiliations:** ^1^​ Department of Infection Genetics, Helmholtz Centre for Infection Research, Braunschweig, Germany; ^2^​ Department of Structure and Function of Proteins, Helmholtz Centre for Infection Research, Braunschweig, Germany; ^3^​ Department of Pathology, University of Veterinary Medicine Hannover, Hannover, Germany; ^4^​ Institute of Virology Muenster (IVM), Westfaelische Wilhelms-University (WWU) Muenster, Muenster, Germany; ^5^​ University of Veterinary Medicine Hannover, Hannover, Germany; ^6^​ Department of Microbiology, Immunology and Biochemistry, University of Tennessee Health Science Center, Memphis, TN, USA

**Keywords:** influenza A virus, host protease, hemagglutinin

## Abstract

The haemagglutinin (HA) of H1N1 and H3N2 influenza A virus (IAV) subtypes has to be activated by host proteases. Previous studies showed that H1N1 virus cannot replicate efficiently in *Tmprss2^−/−^* knock-out mice whereas H3N2 viruses are able to replicate to the same levels in *Tmprss2^−/−^* as in wild type (WT) mice. Here, we investigated the sequence requirements for the HA molecule that allow IAV to replicate efficiently in the absence of TMPRSS2. We showed that replacement of the H3 for the H1-loop sequence (amino acids 320 to 329, at the C-terminus of HA_1_) was not sufficient for equal levels of virus replication or severe pathology in *Tmprss2^−/−^* knock-out mice compared to WT mice. However, exchange of a distant amino acid from H1 to H3 sequence (E31D) in addition to the HA-loop substitution resulted in virus replication in *Tmprss2^−/−^* knock-out mice that was comparable to WT mice. The higher virus replication and lung damage was associated with increased epithelial damage and higher mortality. Our results provide further evidence and insights into host proteases as a promising target for therapeutic intervention of IAV infections.

## Introduction

Influenza A virus (IAV), a member of the *Orthomyxoviridae* family, is responsible for a highly contagious respiratory illness that affects millions of people worldwide during seasonal epidemics causing huge mortality and economic loss [1, 2]. H1N1 and H3N2 are the presently circulating virus subtypes that cause yearly epidemics in humans. The receptor-binding surface molecule haemagglutinin (HA) forms homotrimers [3]. It is synthesized as an inactive precursor molecule HA_0_ and proteolytically processed into the subunits HA_1_ and HA_2_ that are connected by a disulfide bond. Activation of HA is important for its fusion potential with the endosome including a conformational change to release the viral genome into the cytoplasm (reviewed in [4–7]). Depending on the protease recognition motif, several proteases have been identified to cleave HA (reviewed in [5–8]). The transmembrane protease serine 2 (TMPRSS2) has been shown to cleave H1- and monobasic H7-HA *in vitro* at the cleavage sites PSIQSR↓G and PEIPKR↓G, respectively [4, 9]. Furthermore, in mouse knock-out (KO) mutants, it was shown that *Tmprss2-*deficient *(Tmprss2^−/−^)* mice are highly resistant to infections with H1N1 and monobasic H7N9, but only weakly to H3N2 [10–12]. In resistant *Tmprss2^−/−^* mice, H1- and H7-HA are not efficiently cleaved and subsequently the virus fails to spread in infected lungs and therefore does not cause pathology [10–12]. On the other hand, H3N2 is able to replicate in *Tmprss2^−/−^* mice and cause mortality [10, 13]. In *Tmprss2^−/−^ Tmprss4^−/−^* double KO mice, replication of H3N2 is still occurring although at a strongly reduced level resulting in low mortality [14]. Thus, the H3-HA motif PEKQTR↓G is not only processed by TMPRSS2, but also by TMPRSS4 and at least one more, yet unknown protease [14].

In the present study, we aimed to investigate the differences between H1- and H3-HA amino acid sequences that are required for virus replication and pathology in *Tmprss2^−/−^* mice. For this, we exchanged the H3-HA loop sequence for the H1-loop. We showed that in addition to the HA-loop a distant amino acid is necessary for efficient replication and pathogenicity in *Tmprss2^−^/^−^* mice.

## Results

### Generation of recombinant viruses with H3 for H1 amino acid sequence replacements

**Fig. 1.**
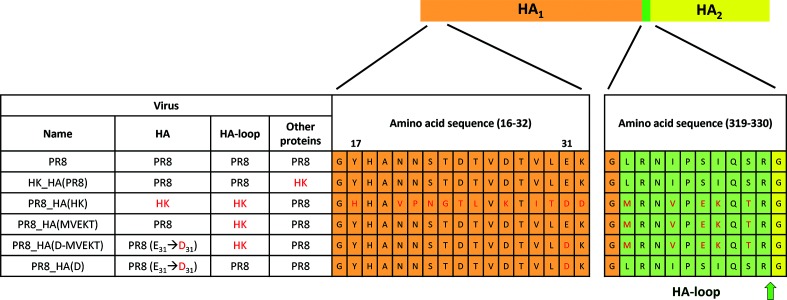
Scheme of recombinant viruses. The structure of recombinant viruses with name, origin of segment 4 (HA), origin of the loop, backbone segments (Other proteins) and relevant HA amino acid sequences are illustrated. Differences at the amino acid level are shown for the N-terminal part (amino acids 16–32) and the protease recognition site with its neighbouring region (amino acids 320–329, H3 numbering), which we refer to here as the ‘HA-loop’ (highlighted in green). HA_1_ is highlighted in orange (except the HA-loop) and HA_2_ in yellow. Differences in amino acid sequences between HA-HK and HA-PR8 are labelled red. The arrow indicates where cleavage occurs. PR8: A/Puerto Rico/8/34 (H1N1); HK: A/Hong Kong/01/68 (H3N2).

In this study we aimed at determining which HA sequences are responsible for the observed differences of virus replication and associated pathology between H1N1 and H3N2 subtypes in *Tmprss2* knock-out *(Tmprss2^−/−^)* mice. For this, we generated several reassortants and mutant viruses based on A/Puerto Rico/8/34 (H1N1) (PR8) and A/Hong Kong/01/68 (H3N2) (HK). Plasmids were generated by sequence and ligation independent cloning (SLIC) and transfected with Lipofectamine 2000 to rescue viruses. We first generated two reassorted viruses, HK_HA(PR8) carrying the entire HA segment of H1 on the backbone of HK, and PR8_HA(HK) carrying the entire HA segment of H3 on the backbone of PR8 (Fig. 1). Furthermore, we generated a recombinant virus [PR8_HA(MVEKT)] in which we replaced the HA cleavage site and neighbouring amino acids of H1-HA with the amino acid sequence of H3-HA (region from amino acids 320 to 329, at the C-terminus of HA_1_, Fig. 1). We refer to this HA region as the ‘HA-loop’ which is predicted to interact with cellular proteases and in which HA cleavage occurs between amino acids R329 and G330 (Fig. 1). Additionally, we produced a virus in which we also exchanged a more distant amino acid from the H1-HA to the H3-HA sequence that is predicted to interact with amino acids in the loop (see below for more details) either by itself or in combination with the loop exchange. In these viruses, referred to as PR8_HA(D) or PR8_HA(D-MVEKT), respectively, the glutamate at position 31 was replaced by aspartate from H3 (E31 to D31, Fig. 1). Of note, we could not rescue recombinant virus with single amino acid exchange in the H1-HA-loop (predicted protease recognition sequence: H1-PSIQSR to H3-PEKQTR) suggesting that the entire H1-HA-loop or H3-HA-loop sequence is required for a functional HA. The replaced and mutated HA segments (segment 4) were incorporated into viruses with an isogenic PR8 backbone for all other segments (Fig. 1).

### HA segment of H1 and H3 determines resistance phenotype in *Tmprss2^−/−^* mice

**Fig. 2.**
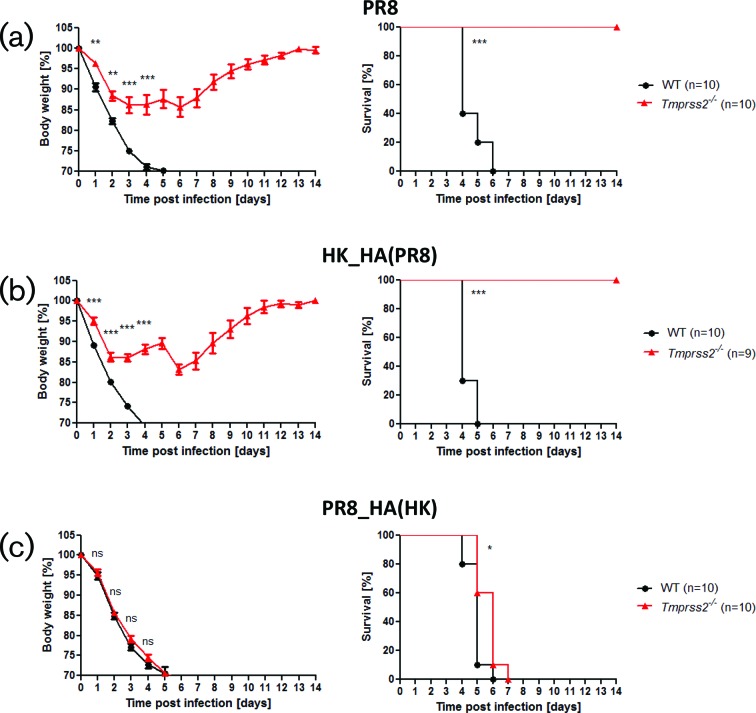
HA determines resistance phenotype in *Tmprss2^−/−^* mice. Female WT and *Tmprss2^−/−^* mice (8–12 weeks old) were infected intranasally with 2×10^5^ f.f.u. of (a) PR8 (H1N1), (b) HK_HA(PR8) (H1N2) or (c) PR8_HA(HK) (H3N1) and body weight was monitored for 14 days p.i. Left: mean body weight in percent of starting weight ±1 SEM. Right: survival graphs. Statistics for body weight loss were performed only for groups in which more than 50 % of infected mice were still alive. Significances were calculated using repeated measures ANOVA followed by a pair-wise *t*-test with Benjamini–Hochberg correction for multiple testing. Statistics for survival curves were calculated with the log rank test. Stars indicate adjusted *P*-values (*P<0.05; **P<0.01; ***P<0.001); ns: non-significant. In addition to mice that were found dead, animals with a body weight loss of more than 30 % of the starting body weight were euthanized and recorded as dead.

WT and *Tmprss2^−/−^* knock-out (KO) mice were infected with 2×10^5^ f.f.u. PR8, HK_HA(PR8) and PR8_HA(HK). After infection with PR8 (H1N1), WT mice lost body weight and all infected mice died, whereas *Tmprss2^−/−^* mice lost body weight only slightly and all infected mice survived (Fig. 2a). Infection with HK_HA(PR8) resulted in body weight loss in WT similar to infections with PR8 whereas *Tmprss2^−/−^* mice showed a similar resistance phenotype as for infections with PR8 (Fig. 2b). These results demonstrate that the resistance phenotype to H1N1 virus in *Tmprss2^−/−^* mice resides in the H1-HA segment and that other proteins of the HK virus do not have a major impact. Infection of *Tmprss2^−/−^* and WT mice with PR8_HA(HK) led to body weight loss in both mouse strains and death of all infected mice (Fig. 2c). These results are consistent with our previous observations using the entire HK (H3N2) virus [10]. Also, results with the reassorted viruses HK_HA(PR8) and PR8_HA(HK) were identical to the results from the parental viruses (PR8, HK) in previous studies when the same infection dose was used (2×10^3^ f.f.u.; Fig. S1, available in the online version of this article). Together, our results demonstrate that exchange of the HA segments leads to a phenotype that resembles that of the parental virus (H1N1 or H3N2, respectively) in *Tmprss2^−/−^* and WT mice. Thus, H3-HA is necessary and sufficient for body weight loss and mortality in *Tmprss2^−/−^* mice.

### Exchange of H1 to H3 cleavage loop is not sufficient for TMPRSS2 escape

**Fig. 3.**
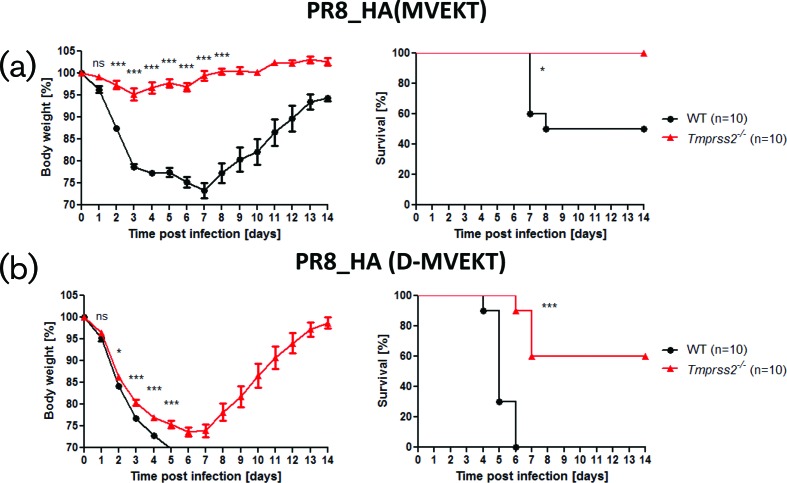
Exchange of the cleavage site and one additional amino acid is required for body weight loss and mortality in *Tmprss2^−/−^* mice. Female WT and *Tmprss2^−/−^* mice (8–12 weeks old) were infected intranasally with 2×10^5^ f.f.u. of (a) PR8_HA(MVEKT), (b) PR8_HA(D-MVEKT) and body weight was monitored for 14 days p.i. Left: mean body weight in percent of starting weight ± 1 SEM. Right: survival graphs. Statistics for body weight loss were performed only for groups in which more than 50 % of infected mice were still alive. Significances were calculated using repeated measures ANOVA followed by a pair-wise *t*-test with Benjamini–Hochberg for multiple testing correction. Statistics for survival curves were calculated with the log rank test. Stars indicate adjusted *P*-values (*P<0.05; ***P<0.001); ns: non-significant. In addition to mice that were found dead, animals with a body weight loss of more than 30 % of the starting body weight were euthanized and recorded as dead.

Next, we sought to identify the specific amino acid sequences in the H3-HA that are responsible for body weight loss and mortality in *Tmprss2^−/−^* mice. For this, we exchanged the H3 sequence (MRNVPEKQTR) for the HA-loop of H1 (LRNIPSIQSR) resulting in virus PR8_HA(MVEKT). Infection of WT mice showed that PR8_HA(MVEKT) was not as pathogenic as the parental PR8 virus; only 50 % of the infected WT mice died after infection with PR8_HA(MVEKT) compared to 100 % lethality of WT mice after infection with PR8 or PR8_HA(HK) (Fig. 3a). More importantly, after infection with PR8_HA(MVEKT) virus, *Tmprss2^−/−^* mice only lost body weight slightly and all infected mice survived (Fig. 3a). These results demonstrate that the exchange of the HA-loop alone from H1-HA to H3-HA is not sufficient to cause strong body weight loss and mortality in *Tmprss2^−/−^* mice, suggesting that additional amino acids outside the HA-loop region are required for this phenotype.

### Additional amino acid exchanges at distant sites allow the resistance to *Tmprss2^−/−^* to be overcome

**Fig. 4.**
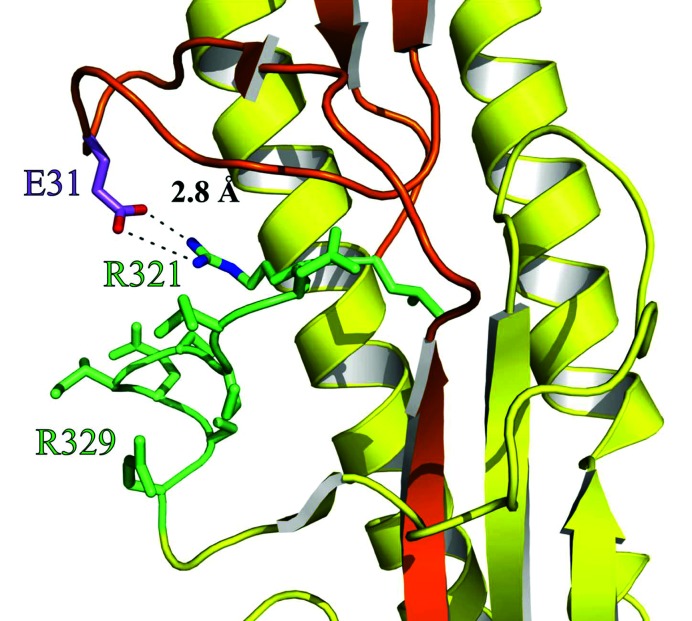
Three-dimensional structure of the cleavage site environment of haemagglutinin H1 suggests interactions with distant amino acids. Details of the haemagglutinin crystal structure from the H1N1 1918 ‘Spanish flu’ virus (PDB ID: 1RD8; [16]) showing HA_1_ in orange, HA_2_ in yellow and the HA-loop (amino acids L320-R329) in green. Residues of the HA-loop including R321 (green) and the adjacent amino acid E31 (purple) are shown as sticks. Additionally, for E31 and R321, nitrogen and oxygen atoms are coloured in blue and red, respectively, and the salt bridge is depicted as the dashed line. The figure was generated with PyMOL [37].

The results described above showed that the exchange of the H3-HA-loop for the H1-HA-loop alone does not result in a virus that overcomes TMPRSS2 dependency. Therefore, we inspected the crystal structure of the H1-HA from the 1918 ‘Spanish flu’ virus (PDB [15] ID: 1RD8; [16]) in more detail. H1-HA of the H1N1 1918 ‘Spanish flu’ virus and the PR8 (H1N1) utilized in this study share a sequence similarity of 89 % and the herein discussed amino acids are present in both proteins. By this approach, we identified a salt bridge formed by arginine at position 321 and glutamic acid at position 31 which is located in proximity to the HA-loop and thus might play a role for protease recognition (Fig. 4).

Therefore, we also replaced this amino acid in H1 with the equivalent amino acid from the H3 sequence in addition to the HA-loop: aspartic acid at position 31 (mutant E31D). This resulted in virus PR8_HA(D-MVEKT) (Fig. 1). Of note, PR8_HA(D-MVEKT) could only be rescued after exchange of the codon GAG (encoding for glutamic acid, nucleotide 144–146) to GAU, but not GAC (both encoding for aspartic acid) which might be due to a restriction in the *cis*-acting specific packaging signal [17]. After infection with PR8_HA(D-MVEKT), *Tmprss2^−/−^* mice showed strong body weight loss with a similar kinetic as WT mice (Fig. 3b). Also, all of the infected WT mice and 40 % of infected *Tmprss2^−/−^* mice died (Fig. 3b). Thus, PR8_HA(D-MVEKT) is able to cause considerable body weight loss and mortality also in *Tmprss2^−/−^* mice although to a lesser extent. We confirmed that both exchanges are required for the higher pathogenicity of PR8_HA(D-MVEKT) by studying the pathogenicity of the single mutant PR8_HA(D) virus (Figs 1 and S2). This virus exhibits slightly higher pathogenicity (body weight loss similar, one mouse died) in *Tmprss2^−/−^* mice than PR8 but was much less virulent than PR8_HA(D-MVEKT).

In conclusion, these results demonstrate that both the loop and distant amino acids are necessary for causing severe symptoms upon infection in *Tmprss2^−/−^* mice.

### Exchange of the HA-loop and a distant amino acid to H3 increases viral replication in *Tmprss2^−/−^ mice*

**Fig. 5.**
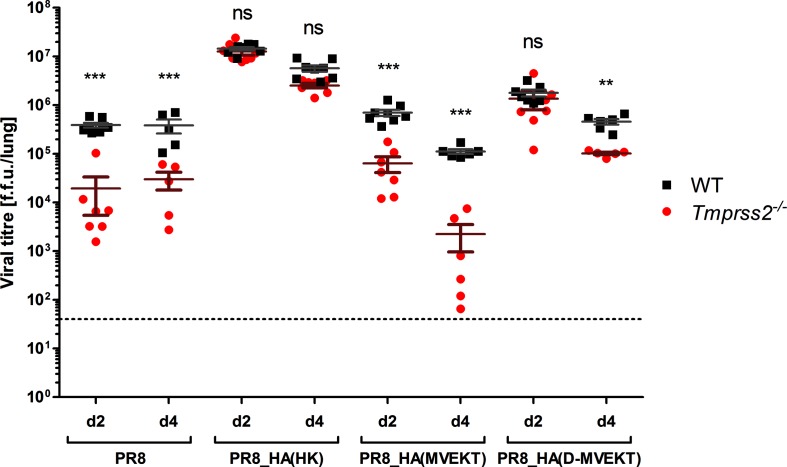
PR8_HA(D-MVEKT) but not PR8_HA(MVEKT) virus replicates to high titres in *Tmprss2^−/−^* mice. Female 8–12-week-old WT and *Tmprss2^−/−^* mice were infected intranasally with 2×10^5^ f.f.u. PR8 (d2: WT *n*=8, KO *n*=7; d4: WT *n*=5, KO *n*=5), PR8_HA(HK) (d2: WT *n*=7, KO *n*=8; d4: WT *n*=8, KO *n*=7), PR8_HA(MVEKT) (d2: WT *n*=8, KO *n*=7; d4: WT *n*=6, KO *n*=6) and PR8_HA(D-MVEKT) (d2: WT *n*=7, KO: *n*=7; d4 WT *n*=6, KO *n*=5). On day 2 and 4 p.i., lungs were homogenized and titres were determined by f.f.u. in each lung (each sample was titrated 2–4 times). Mean titres are shown with ± 1 SEM. Statistical significance was determined by a multi-factorial ANOVA and post-hoc t-test with Benjamini-Hochbertg correction for multiple testing. Stars indicate statistical significane results for treatment groups between WT and *Tmprss2^−/−^* mice: *P*-values (**P<0.01, ***P<0.001); ns: non-significant.

Next, we investigated if the higher mortality in PR8_HA(D-MVEKT)-infected mice was due to increased viral replication. WT and *Tmprss2^−/−^* mice were infected with 2×10^5^ f.f.u. PR8, PR8_HA(HK), PR8_HA(MVEKT) and PR8_HA(D-MVEKT). On day 2 and 4 p.i., viral loads were determined in infected lungs (Fig. 5). ANOVA of the viral load as the response variable revealed significant effects of virus-type and mouse-strain as well as their interaction. Pair-wise comparisons showed that viral loads were significantly higher in WT compared to *Tmprss2^−/−^* mice after infection with PR8 and PR8_HA(MVEKT) at 2 and 4 days p.i. demonstrating that viral replication is strongly reduced in the absence of TMPRSS2 (Fig. 5). On the other hand, no significant difference was observed between WT and KO mice after infection with PR8_HA(HK) and PR8_HA(D-MVEKT) on day 2 suggesting that replacement of the HA-loop plus a distant amino acid allows efficient replication of PR8_HA(D-MVEKT) in *Tmprss2^−/−^* mice (Fig. 5). At day 4 after infection with HK_HA(PR8) the viral load was lower than at day 2, but still the same for WT and *Tmprss2^−/−^* (Fig. 5). In contrast to this observation, the viral load of PR8_HA(D-MVEKT) at 4 days p.i. was lower in *Tmprss2^−/−^* mice compared to the WT. This is in agreement with the higher survival rate of *Tmprss2^−/−^* mice. Additionally, viral replication was significantly higher in *Tmprss2^−/−^* mice infected with PR8_HA(D-MVEKT) compared to infections with PR8_HA(MVEKT) or PR8 at day 2 and 4 (Fig. 5). Thus, the difference in virus virulence (body weight loss and mortality) between PR8_HA(D-MVEKT) and PR8_HA(MVEKT) can be explained by differences in viral replication in infected lungs and thus increased tissue damage.

### Increased lung pathology in viruses with loop and distant amino acid replacements

**Fig. 6.**
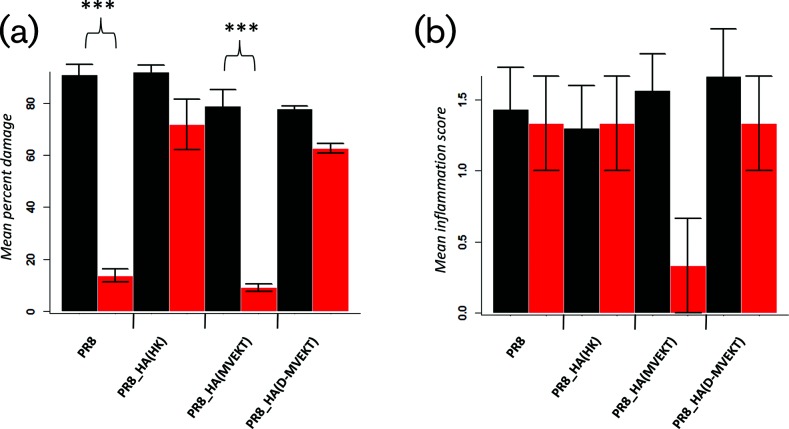
PR8_HA(D-MVEKT) but not PR8_HA(MVEKT) virus causes epithelial damage in *Tmprss2^−/−^* mice. Female 8–12-week-old WT and *Tmprss2^−/−^* KO mice were infected intranasally with 2×10^5^ f.f.u. PR8 (WT: *n*=3; KO: *n*=3), PR8_HA(HK) (WT: *n*=3; KO: *n*=3), PR8_HA(MVEKT) (WT: *n*=3; KO: *n*=3) or PR8_HA(D-MVEKT) (WT: *n*=3; KO: *n*=3). Lungs were prepared at day 4 p.i., sectioned and stained with H&E. Bronchiolar epithelial damage (a) and inflammation (b) were scored. WT mice: black, *Tmprss2^−/−^* mice: red. Error bars represent ±1 sem. Statistical significance was determined by ANOVA and a post-hoc pair-wise *t-*test and Bonferroni correction for multiple testing. *P*-values of <0.05 were considered significant (***P<0.001). Inflammation scores were not significantly different by ANOVA. Therefore, no post-hoc analysis was performed.

**Fig. 7.**
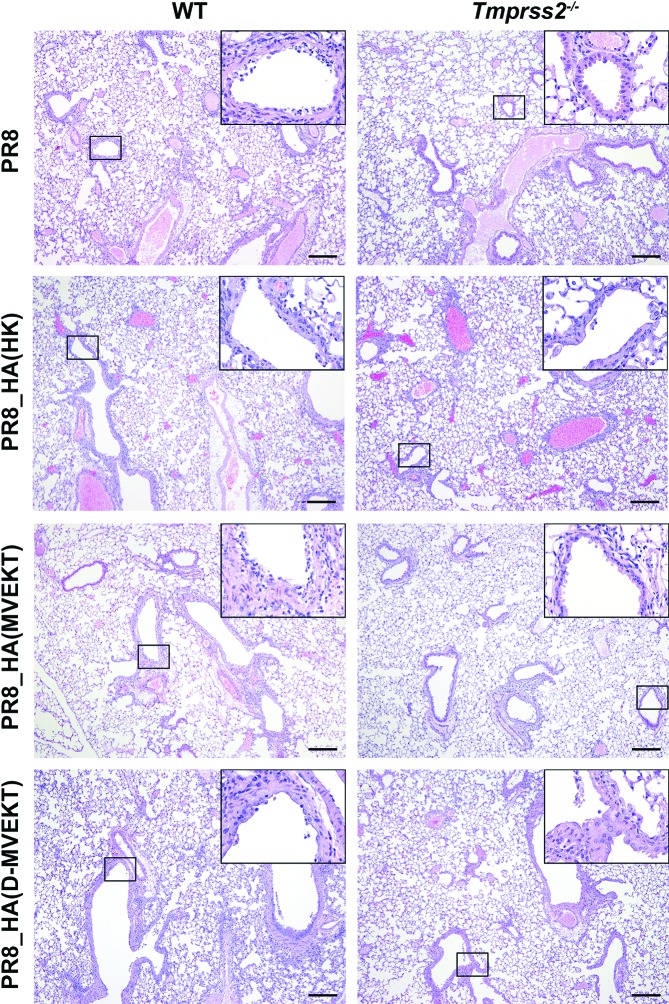
PR8_HA(D-MVEKT) but not PR8_HA(MVEKT) virus infection results in severe bronchiolar epithelial damage in *Tmprss2^−/−^* mice. Female 8–12-week-old WT and *Tmprss2^−/−^* KO mice were infected intranasally with 2×10^5^ f.f.u. PR8 (WT: *n*=3; KO: *n*=3), PR8_HA(HK) (WT: *n*=3; KO: *n*=3), PR8_HA(MVEKT) (WT: *n*=3; KO: *n*=3) and PR8_HA(D-MVEKT) (WT: *n*=3; KO: *n*=3). Lungs were prepared at day 4 p.i., sectioned and stained with H&E. PR8 and all recombinant viruses caused severe damage of bronchial epithelium (inserts) in the lungs from WT mice. In contrast, in *Tmprss2^−/−^* mice regions of severely damaged epithelial layers (inserts) were mainly observed in PR8_HA(HK) and PR8_HA(D-MVEKT) infected animals, whereas PR8 and PR8_HA(MVEKT) only produced mild or no damage of bronchial epithelium (inserts). Note also the similar degree of inflammatory cell infiltration in all infected mice. Bars, 200 µm.

To investigate if increased viral replication of the PR8_HA(D-MVEKT) virus was associated with increased lung tissue damage, we performed histopathological studies of infected lungs. After infection with PR8_HA(MVEKT) and PR8, lungs showed significantly reduced bronchiolar epithelial damage in *Tmprss2^−/−^* mice compared to WT mice (Fig. 6a). On the other hand, epithelial damage scores were equally high (no significant difference) for *Tmprss2^−/−^* mice and WT mice after infection with PR8_HA(HK) and PR8_HA(D-MVEKT) (Fig. 6a). By ANOVA, no significant differences were observed for inflammation scores (Fig. 6b). Fig. 7 shows representative sections after infection of WT or *Tmprss2^−/−^* mice with PR8, PR8_HA(HK), PR8_HA(MVEKT) and PR8_HA(D-MVEKT). In conclusion, the difference in virulence (body weight loss and mortality) between PR8_HA(D-MVEKT) and PR8_HA(MVEKT) is due to an increase in tissue damage resulting from higher levels of viral replication.

### Cleavage of HA is correlated with exchange of the HA-loop

**Fig. 8.**
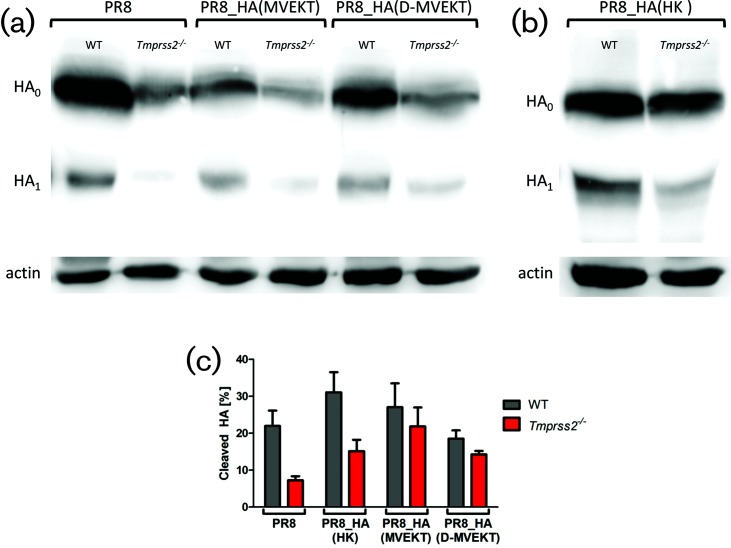
The HA of PR8_HA(MVEKT) and PR8_HA(D-MVEKT) viruses was cleaved in lungs of infected *Tmprss2^−/−^* mice. Female 8–12-week-old WT and *Tmprss2^−/−^* mice were infected intranasally with 2×10^5^ f.f.u. PR8, PR8_HA(MVEKT), and PR8_HA(D-MVEKT), and PR8_HA(HK). On day 2 p.i., lungs were homogenized and total protein was quantified. For each sample, 50 µg total protein was run on a SDS-PAGE, blotted to a PVDF membrane and stained by (a) an anti-H1N1 antibody (PR8) or (b) an anti-H3N2 antibody (A/Brisbane/10/2007) and both with a HRP-conjugated goat anti-rabbit antibody. HA_0_: uncleaved HA, HA_1_: N-terminal part of cleaved HA. Detection of signals was performed using the FujiFilm LAS-3000 imaging system. Subsequently, the same membrane was incubated with mouse anti-actin, followed by incubation with HRP-conjugated horse anti-mouse IgG and visualized again. Corresponding actin bands are shown below each sample. (c) Cleaved HA is shown as the ratio of HA1*100/(HA1+HA0). Three blots derived from the same lung homogenate samples were analysed using ImageJ software and the mean was depicted ±1 SEM.

Finally, we investigated cleavage of HA in lung homogenates on day 2 p.i. after infection with PR8, PR8_HA(HK), PR8_HA(MVEKT) and PR8_HA(D-MVEKT) (Fig. 8). In line with our previous results, HA of PR8 was cleaved in WT mice, but only slightly in *Tmprss2^−/−^* mice (Fig. 8a). The difference to previously published data is most likely due to a higher infection dose used in this study [10]. Out of three blots of the same lung samples, the ratio between HA_1_ to HA_1_+HA_0_ was calculated and the mean depicted (Fig. 8c). After infection with PR8, 22 % of HA was cleaved in WT mice, but only 7  % in *Tmprss2^−/−^* mice. As described above, HA of HK was cleaved in WT (31 %) and *Tmprss2^−/−^* mice (15  %) (Fig. 8b). Likewise, the HA of PR8_HA(D-MVEKT) was cleaved in both WT (19  %) and *Tmprss2^−/−^* mice (14  %), which correlates with its high replication rate and pathogenicity (Fig. 8a). These results were confirmed in the Western blots of viral proteins from bronco-alveolar lavages (BAL; Fig. S3).

In conclusion, the exchange of the H3-HA-loop alone for the H1-HA-loop allows for HA cleavage but does not result in high viral replication or pathology (survival, body weight loss, lung damage) in infected *Tmprss2^−/−^* mice. Exchange of the HA-loop and an additional amino acid is required for high level of replication and lung pathology in *Tmprss2^−/−^* mice.

## Discussion

We and others have previously shown that deletion of *Tmprss2* in mice results in resistance to infection with H1N1 and monobasic H7N9 IAV [10–12]. Yet, two studies showed that replication of H3N2 was only marginally affected and caused pathology in *Tmprss2^−/−^* mice [10, 12]. A third study described an H3N2 variant that was not able to replicate in *Tmprss2^−/−^* mice [11]. However, after serial passages in *Tmprss2^−/−^* mice, this virus acquired a mutation in the HA stalk which led to replication and pathology in *Tmprss2^−/−^* mice [13]. Moreover, we recently demonstrated that in *Tmprss2^−/−^ Tmprss4^−/−^* double knock-out mice, replication and pathology after infection with our H3N2 isolate was strongly diminished and infected mice survived. These observations suggest that H3 viruses are able to replicate in a *Tmprss2^−/−^* background whereas H1 viruses cannot. Here, we investigated in detail the differences in the HA sequence that are responsible for the different behaviour of H1 and H3 viruses in *Tmprss2^−/−^* mice. For this, we generated a series of reassorted and mutated IAV and analysed body weight loss, survival, viral replication in lung, lung epithelial cell damage, and cleavage of the HA protein in WT and *Tmprss2^−/−^* mice.

We first established that the exchange of only the HA segment of H3-HA for H1 in a virus that contained all other segments from PR8 (H1N1) resulted in 100 % lethality in *Tmprss2^−/−^* mice [virus PR8_HA(HK)]. *Vice versa*, replacement of the H3-HA by the H1-HA in an H3N2 background completely protected infected *Tmprss2^−/−^* mice from body weight loss and mortality [virus HK_HA(PR8)]. These studies confirmed that the resistance phenotype in *Tmprss2^−/−^* mice to H1N1 IAV only resides in the HA protein. This important aspect had not been addressed in any of the previous studies [10–12].

The replacement of only the HA-loop was not sufficient to overcome the resistant phenotype of *Tmprss2^−/−^* mice. However, replacement of the HA-loop and additional replacement of a distant amino acid, E31 by aspartic acid from H3-HA, strongly increased mortality of *Tmprss2^−/−^* mice after infection [virus PR8_HA(D-MVEKT)]. The increase in pathology was due to a higher viral replication and increased lung epithelial damage which was comparable to WT mice infected with this virus. E31 is predicted to interact with R321 of the HA-loop in H1-HA. The exchange of E31 and accompanied changes of the HA protease recognition site could lead to an altered accessibility of the HA-loop for other proteases than TMPRSS2 in [PR8_HA(D-MVEKT)]. Several *in vitro* studies also suggested that the exchange of the HA-loop itself does not necessarily lead to productive HA processed by matriptase protease [18]. Furthermore, when the cleavage site of an H1-HA was exchanged by the amino acid sequence RSSR, matriptase was still unable to cleave the HA-loop. Similarly, an H9-HA carrying the minimal furin cleavage motif was not cleaved by furin [19]. Further studies are thus needed to address the role of additional proteases and requirements for a fully functional HA processing. The importance of a distant amino acid on the pathogenicity of an H3 virus had been shown before by exchanging amino acid 8 in HA resulting in the loss of a glycosylation site and increased pathogenicity [13, 20]. In addition, it was shown that cleavage by TMPRSS2 results in a less glycosylated HA_1_ compared to cleavage by trypsin or TMPRSS4 [8]. Thus, our *in vivo* study confirms previous *in vitro* analyses demonstrating the role of loop and distant amino acids for viral replication.

However, it should be noted that the above changes were still not sufficient to completely restore the high virulence of a virus with a complete H3-HA [PR8_HA(HK)], suggesting that further distant amino acids might be important as well.

Also, it should be noted that any change in the amino acid sequence, which was introduced in this study, may have an influence on viral fitness and thus replication rate and pathogenicity. This can be clearly seen when the replication of different viruses was compared in WT mice. However, in this study, we did not seek to investigate the effect of these amino acid exchanges on viral fitness in WT mice but our study aimed to understand the role of specific amino acids in the loop and potentially interacting amino acids on the dependency of virus replication and pathogenicity for the TMPRSS2 protease. Thus, our main conclusions relate to the difference of virus pathogenicity and replication between WT and *Tmprss2^−/−^* mice. We showed that amino acids of the loop together with a distant amino acid are important for cleavage by proteases other than TMPRSS2.

Many studies performed in the mouse model system demonstrated that virus replication in the lung and associated tissue damage or functional impairment of infected cells is the main cause for severe pathology [21, 22]. In other studies, the degree of the inflammatory response and the resulting immunopathology has also been described as a major contributor to disease severity [21, 23]. In this study, histopathological analysis showed that tissue damage was the major correlate for disease severity. It also correlated well with replication (between WT and mutant mice for a given virus) whereas tissue inflammation was not significantly correlated. We thus hypothesize that virus replication and subsequent tissue damage is the main cause for disease severity. This conclusion is well in line with the interpretation that the absence or presence of specific proteases is the main driver for virus maturation, and subsequently its replication and spread in the infected lung and eventually severity of the disease. A similar result was obtained from patients infected with pandemic 2009 H1N1 or H7N9. Patients whose genome carried a higher-expression variant of TMPRSS2 were more severely ill than patients expressing less TMPRSS2 [24].

A simple explanation for the reduced viral titres and spreading of virus PR8_HA(MVEKT) and the increased replication of virus PR8_HA(D-MVEKT) in *Tmprss2^−/−^* mice would be the absence or reduction of HA cleavage in PR8_HA(MVEKT). However, Western blot analysis revealed that the HA-loop of both viruses, PR8_HA(MVEKT) and PR8_HA(D-MVEKT), was cleaved in *Tmprss2^−/−^* mice. Nevertheless, PR8_HA(MVEKT) was less efficiently cleaved compared to PR8_HA(D-MVEKT). One likely explanation could be that PR8_HA(MVEKT) is cleaved by a protease that recognizes the HA protein at a nearby but markedly different cleavage site than the trypsin-like TMPRSS2. Such a shift by a single or three amino acids is sufficient to cause cleavage but would thus not result in efficiently activated particles as has been reported for cleavage of H3-HA by thermolysin and chymotrypsin [25, 26]. A second possibility could be that the respective unknown protease cleaves at the same site as TMPRSS2 but does not generate activated particles, as has been described for hepsin [8]. In both cases, these viruses would not be infectious and do not lead to high titres, tissue damage and death of the animals although the HA protein is cleaved.

Which other protease(s) could be responsible for the cleavage of HA(D-MVEKT) in *Tmprss2^−/−^* mice? We showed recently that in addition to TMPRSS2 also TMPRSS4 was required for optimal cleavage and replication of H3-HA [14]. Another candidate may be TMPRSS11D (HAT), which was shown *in vitro* to cleave H3-HA [27, 28]. Additionally, it was described that H3 can be cleaved *in vitro* by kallikrein-related peptidase 5 (KLK5), tryptase Clara (TPSB2), TMPRSS11E (DESC1) and TMPRSS13 (MSPL) [6, 29, 30]. For these studies, additional mouse mutants with single and double KO will be required. Recently, a *Klk5*-deficient knock-out mouse was described that showed no difference in body weight loss compared to WT mice after infection with a H3N2 IAV supporting again the hypothesis that more than one protease is responsible for cleavage of H3-HA [31]. To identify the third protease that cleaves H3-HA *in vivo*, additional mouse mutants with single, double or even triple KO will be required.

In conclusion, exchanging the H3-HA sequences for the H1-HA-loop plus one distant amino acid increases virus replication and pathology in *Tmprss2^−/−^* mice. More studies are still needed to fully understand these processes and identify yet unknown proteases as well as to eventually develop inhibitors against host proteases that can be used for intervention treatment in humans. Nevertheless, our results provide further support for host proteases as a highly potential target to inhibit IAV replication and pathology.

## Methods

### Cells and viruses

Madin-Darby canine kidney II (MDCK) cells (ATCC) and human embryonic kidney (HEK) 293T cells (Open Biosystem) were kept at 37 °C with 5  % CO_2_ in minimal essential medium (MEM, Gibco) containing 10  % fetal calf serum (PAA Laboratories) and 1  % Penicillin/Streptomycin (P/S, Biochrom). A/Hong Kong/01/68 (H3N2) was originally obtained from Otto Haller, University of Freiburg. All viruses were propagated in the chorio-allantoic cavity of 10-day-old specific pathogen free (SPF) embryonated chicken eggs (Charles River Laboratories, Germany) for 48 h at 37 °C, aliquoted and stored at −80 °C. The titre of the stock viruses was determined by f.f.u. assay (f.f.u. ml^–1^). Viral RNA was extracted using the QiAamp Viral RNA Extraction Kit (Qiagen) according to the manufacturer’s protocols. Quality and integrity of total RNA was controlled on Agilent Technologies 2100 Bioanalyzer (Agilent Technologies; Waldbronn, Germany). The RNA sequencing library was generated from 100 ng total RNA using Illumina True Seq RNA Sample Prep Kit (Illumina) without fragmentation according to the manufacturer’s protocols. The libraries were sequenced on Illumina MiSeq using MiSeq Reagent Kit v2 (500 cycles, paired end run).

### Infection of mice

Original *Tmprss2*-mutant mice were kindly provided by Peter S. Nelson, FHCRC, Seattle, USA [32]. Exons 10–13, essential for serine protease activity, were deleted through homologue recombination with the targeting vector. Mice were on a mixed background and further backcrossed in our facility to C57BL/6JRj (Janvier, France) for nine successive generations (B6.129S1-*Tmprss2*^tm1Tsyk^). The correct background was verified by Neogen MegaMUGA (High Density Mouse Universal Genotyping Array) SNP-Assay. The analysis demonstrated 99.79 % matching to the C57BL/6J genome with a remaining 8.9 Mb region flanking the original 129-*Tmprss2* genomic region on chromosome 16. C57BL/6JRj WT mice used as controls were purchased from Janvier (France) and maintained under SPF conditions at the Central Animal Facilities at the HZI, Braunschweig. Mice (female, 8–12 week old) were anesthetized by intra-peritoneal injection of Ketamine-Xylazine solution (5 mg ml^−1^ Ketamine, WDT, Garbsen; 1 mg ml^−1^ Xylazine, CP-Pharma, Burgdorf; in sterile 0.9  % NaCl, WDT, Garbsen) with a dose adjusted to the individual body weight (200 µl/20 g body weight). Virus was diluted in sterile PBS to a dose of 2×10^5^ f.f.u./20 µl and mice were infected by intranasal application. Subsequently, body weight and survival were monitored for 14 days. In addition to mice that were found dead, animals with a body weight loss of more than 30  % of the starting body weight were euthanized and recorded as dead. All mouse infections were performed in at least two independent experiments.

### Sequence and ligation independent cloning and mutagenesis

For cDNA preparation, the Uni12 primer (1 µl, 10 mM) was bound to 500 ng viral RNA with 1 µl dNTP in a total volume of 13 µl at 65 °C for 5 min and cooled on ice for 1 min. Then, 4 µl 5x First-Strand Buffer, 1 µl 0.1 M DTT, 1 µl RNasin (Promega) and 1 µl SuperScriptIII (Invitrogen) were added. Reverse transcription was performed for 50 min at 50 °C and stopped for 15 min at 70 °C. Finally, RNA was digested using 1 µl RNaseH (Invitrogen) for 20 min at 37 °C. Plasmids originated from A/Hong Kong/01/68 (H3N2) were cloned into pHW-2000 vector [kindly provided by Robert Webster (St. Jude, Memphis, USA)] by SLIC modified from [33]. First, a PCR was performed using 1 µl PRECISOR High-Fidelity DNA Polymerase (BioCat) on 0.5 µl cDNA with 2 µl of each primer (10 µM) and 1.25 µl dNTPs (10 mM each) in a total of 50 µl, running the program 98 °C 2 min; 35×98 °C 30 s, 57–60 °C 30 s, 72 °C 1–4 min; 72 °C 10 min in a thermocycler. Empty vector pHW-2000 was cut with BsmBI (NEB) for 2 h at 55 °C. Samples were run on an agarose gel and bands of appropriate size were cut and purified using NucleoSpin Gel and PCR Clean-up kit (Macherey-Nagel). Next, 5′ overhangs were made using 50 ng cut vector and PCR product in a 1 : 6 ratio with 0.1 µl T4 DNA polymerase (NEB) and 1 µl buffer 2.1 in a total of 10 µl at 22 °C for 30 min, heat inactivated at 75 °C for 20 min and finally annealed at 37 °C for 30 min. Last, 5 µl of this reaction was transformed into 100 µl chemically competent XL1-Blue cells (Stratagene). To insert mutations into the HA, PCR was performed using 1.5 µl PfuUltra High-Fidelity DNA Polymerase (Stratagene), 5 µl 10x PfuUltra buffer, 1 µl dNTPs (10 mM each), 50 ng plasmid and 1 µl of each primer (10 µM) in a total volume of 50 µl. The following program was run in a thermocycler: 2 min 95 °C; 18×30 s 95 °C; 30 s 55 °C and 5 min 72 °C; 5 min 72 °C and cooled down to 4 °C. The pHW-HA was digested with 1 µl DpnI at 37 °C for 1 h and then chemically competent XL1-Blue cells were transformed using the final product. Plasmid DNA was purified with the Roti-Prep Plasmid MINI (Roth) according to the manufacturer’s instructions. Correct insertion of the mutation was verified by sequencing. Primer lists for mutagenesis and cloning are provided in the online Supplementary Material.

### Generation of reassortant viruses by reverse genetics

Recombinant viruses were generated by DNA transfection as described earlier [34]. All pHW-2000 plasmids containing eight segments of PR8 (A/Puerto/Rico/8/34, H1N1) were kindly provided by Robert Webster (St. Jude, Memphis, USA). HEK 293T and MDCK cells were co-cultured in a six-well plate and grown 24 h at 37 °C in 5  % CO_2_ until cells reached confluency. Cells were washed and 800 µl OptiMEM (Gibco) were added. Transfection was performed using Lipofectamine 2000 (Invitrogen) or *Trans*IT-LT1 (Mirus Bio LLC) according to the manufacturer’s instructions using 1 µg of each plasmid and 15 µl Lipofectamine or 16 µl *Trans*IT-LT1, respectively. Within 24 h post transfection medium was exchanged with 2 ml MEM containing 1 % P/S and 1 µg TPCK-trypsin (tosyl phenylalanyl chloromethyl ketone treated trypsin) (Sigma). Cells were monitored every day until a cytopathic effect appeared and virus was harvested. After amplification of virus, RNA was extracted and whole virus genome was confirmed using next generation sequencing by Illumina (see Cells and viruses).

### Preparation of lung homogenates and virus titration

Lungs were homogenized using FastPrep-24 Instrument (MP Biomedicals). For this, 2ml PBS/0.1  % bovine serum albumin (BSA, Sigma-Aldrich) was added to the lungs in a Lysing MatrixD tube (MP Biomedicals) and homogenized for 30 s at 6.5 m s^−1^. Cell debris was spun down for 10 min with 200***g*** at 4 °C to obtain the supernatant containing virus. Virus titres were determined using f.f.u. assay. For this, viruses were serially tenfold diluted in infection medium [MEM containing 0.1 % BSA, 2.5 µg ml^−1^ NAT (N-acetylated trypsin, Sigma-Aldrich) and 1 % P/S]. MDCK II cells (6×10^4^ cells/well of a 96-well plate, grown for 24 h in MEM supplemented with 1 % P/S) were washed with infection medium. Afterwards cells were infected with 50 µl of each virus dilution. After 1 h of incubation at 37 °C in 5  % CO_2_, cells were overlayed with 100 µl DMEM containing 1  % avicel, 2 mM l-Glutamine, 0.1  % BSA and 2.5 µg ml^−1^ NAT and kept at 37 °C in 5  % CO_2_. Cells were stained 24 h p.i. at room temperature. For this, cells were washed with 100 µl PBS and fixed with 96  % ice-cold ethanol for 10 min. Subsequently, cells were washed twice with 100 µl PBS and permeabilized with 0.5  % Triton X-100 and 20 mM glycin in PBS for 10 min. Cells were washed with 100 µl wash buffer (WB) (0.5  % Tween 20 in PBS) and 50 µl primary antibody were added for 40 min (1 : 1000 in WB, goat anti-influenza virions 1301, Virostat). After two wash steps with 100 µl WB, 50 µl of the secondary antibody were added for 40 min (1 : 1000 in WB, anti-goat-IgG-HRP, KPL, MA, USA). Finally, after two wash steps with 100 µl WB, 30 µl of the substrate were added (TrueBlue, KPL) and incubated for 30 min until blue spots appeared. The substrate was then washed with tap water, cells were dried and foci were counted. Titres were calculated as f.f.u. ml^−1^ (virus stocks) or f.f.u./lung (lung homogenates).

### Histopathology

Lungs were prepared from infected mice and immersion-fixed for 24 h in 4  % buffered formaldehyde solution (pH 7.4), dehydrated in a series of graded ethanol and embedded in paraffin. Sections were cut from five evenly distributed levels and stained with haematoxylin and eosin. For each group (KO and WT, virus), three mice were studied and five sections per mouse were analysed for inflammation using a semi-quantitative scoring system from 0=none, 1=mildly, 2=moderately, 3=severely increased cellular infiltration. In addition, bronchiolar structures with and without epithelial cell necrosis were counted on one section to calculate percentages of affected bronchi.

### Western blots

Total protein was measured in lung homogenates (2 days p.i.) or BAL samples (3 days p.i.) using the Pierce BCA Protein Assay Kit (Thermo Scientific) according to the manufacturer’s instructions. Samples were then mixed with 8x SDS loading buffer and 50 µg total protein in lung homogenates or 20 µg total protein in BAL of each sample was run on a 10  % SDS gel. Proteins were blotted on a PVDF-membrane (Bio-Rad) and H1 was detected by staining with rabbit anti-H1N1-HA antibody (PR8, Sino biological, 11684-RP01), H3 after staining with rabbit anti-H3N2-HA (A/Brisbane/10/2007, Sino biological, 11056-PR02) or NP protein with anti-NP antibody (GeneTex, GTX125989), all at a dilution of 1 : 5000, followed by incubation with HRP-conjugated goat anti-rabbit antibody in a dilution of 1 : 10 000 (Sigma, A0545). Bands were visualized using Western Chemiluminescent HRP substrate (Immobilon) in an imaging system (FujiFilm LAS-3000). The blot with lung homogenates was then incubated with mouse anti-actin (Santa Cruz, sc-47778) diluted 1 : 1000, followed by incubation with HRP-conjugated horse anti-mouse IgG (Cell signaling, 7076) diluted 1 : 3000 and visualized as described before. Intensities of bands were analysed using ImageJ [35].

### Statistical analysis

Data and statistical analysis were performed using GraphPad Prism 5.0 (GraphPad Software, CA) and R software package [36]. Statistical significance for body weight loss was determined by a multi-factorial ANOVA (including repeated measures for mice as subject) using statistical software R and the model body_weight ~ mouse_strain * days_pi + (1|subject) and post-hoc pair-wise *t*-test with Benjamini–Hochberg multiple testing correction. For the ANOVA, only the days p.i. were considered when at least 50  % of infected mice were still alive (2×10^5^ f.f.u.: PR8: days p.i. 1 to 4; HK_HA(PR8): days p.i. 1 to 4; PR8_HA(MVEKT): days p.i. 1 to 8; PR8_HA(D-MVEKT): days p.i. 1 to 5; PR8_HA(HK): days p.i. 1 to 4; PR8_HA(D): days p.i. 1 to 5; 2×10^3^ f.f.u.: PR8_HA(HK): days p.i. 1 to 6; HK_HA(PR8): days p.i. 1–6). Statistical significance for viral load was determined by a multi-factorial ANOVA using statistical software R and the model lg10_virus_titre ~ virus_type * mouse_strain. Statistical significance for lung pathology was determined by a multi-factorial ANOVA using statistical software R and the model mean_inflammation_score ~ group and percent_damage ~ group. *P*-values of <0.05 were considered significant. GraphPad Prism was used to calculate statistical differences for survival curves with the Log-rank (Mantel–Cox) test.

## Supplementary Data

Supplementary File 1
