# Supplementary material for: Exchange of amino acids in the H1-haemagglutinin to H3 residues is required for efficient influenza A virus replication and pathology in Tmprss2 knock-out mice
Source: J Gen Virol. 2018 Aug 6;99(9):1187–98. doi: 10.1099/jgv.0.001128 (PMC6230768; doi:10.1099/jgv.0.001128)
Supplement: Supplementary File 1 [file jgv-99-1187-s001.pdf]

Supplements

Exchange of amino acids in the H1-hemagglutinin to H3 residues is required for efficient influenza A virus replication and pathology in Tmprss2 knock-out mice

Ruth L.O. Lambertz[1], Jan Pippel[2], Ingo Gerhauser[3], Heike Kollmus[1], Darisuren Anhlan[4], Eike R. Hrincius[4], Joern Krausze[2], Nora Kühn[1], Klaus Schughart[1,5,6,#]

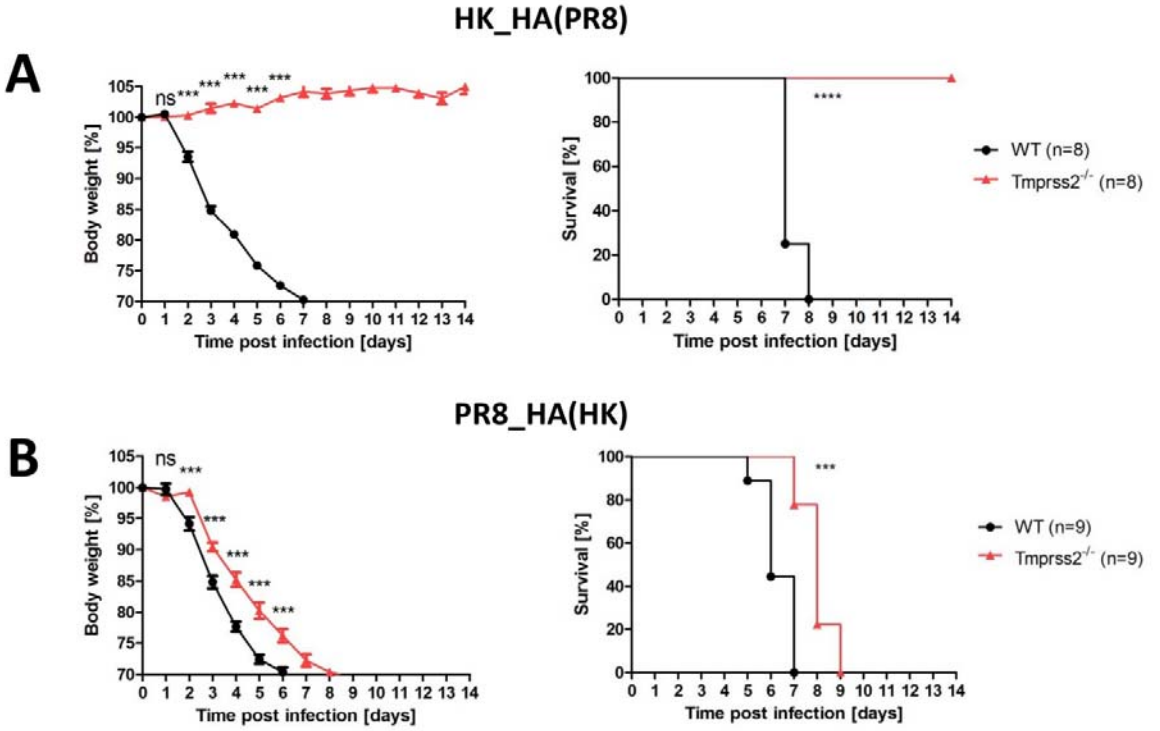

FIG S1: HA determines resistance phenotype in *Tmprss2*$^{-/-}$ mice – lower dose (2x10^{3}ffu) infection

Female wild type (WT) and *Tmprss2*$^{-/-}$ mice (8-12 weeks old) were infected intranasally with 2x10^{3}ffu of (A) HK_HA(PR8) (H1N2), (WT: n=8; KO: n=8) or (B) PR8_HA(HK) (H3N1) (WT: n=9; KO: n=9) virus and body weight was monitored for 14 days p.i. Left panel: Mean body weight in percent of starting weight ± 1 SEM. Right panel: Survival graphs. Statistics for body weight loss

were performed only for groups in which more than 50 % of infected mice were still alive. Significances were calculated using repeated measures ANOVA followed by a pair-wise t-test with BH for multiple testing correction. Statistics for survival curves were calculated with the log rank test. Stars indicate adjusted p-values *** p<0.001; **** p<0.0001); ns: non-significant. In addition to mice that were found dead, animals with a body weight loss of more than 30 % of the starting body weight were euthanized and recorded as dead.

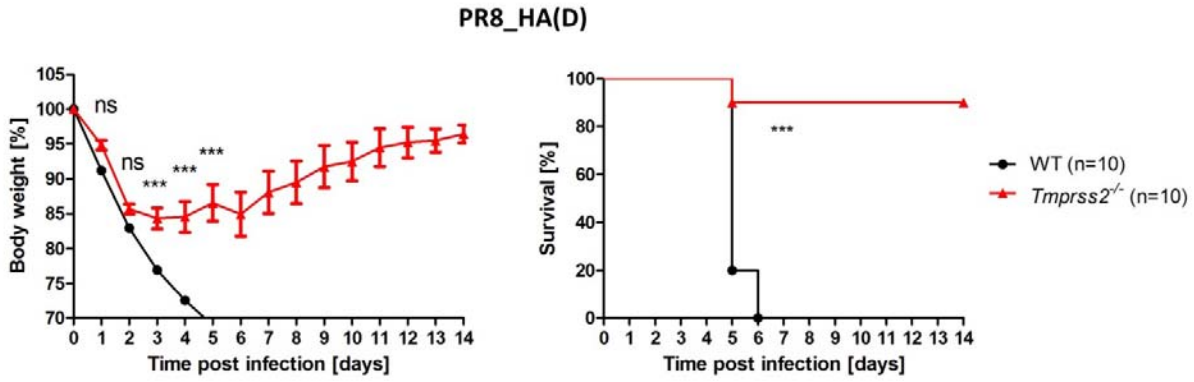

FIG S2: Single amino acid exchange in PR8_HA(D) virus does not result in high pathogenic phenotype

Female wild type (WT, n=10) and *Tmprss2*$^{-/-}$ (n=10) mice (8-12 weeks old) were infected intranasally with $2x10^5$ ffu PR8_HA(D) virus and body weight was monitored for 14 days p.i.. Left Panel: Mean body weight in percent of starting weight ± 1 SEM. Right panel: Survival graphs. Statistics for body weight loss were performed only for groups in which more than 50 % of infected mice were still alive. Significances were calculated using repeated measures ANOVA followed by a pair-wise t-test with BH for multiple testing correction. Stars indicate adjusted p-values (*** p<0.001); ns: non-significant. In addition to mice that were found dead, animals with

a body weight loss of more than 30 % of the starting body weight were euthanized and recorded as dead.

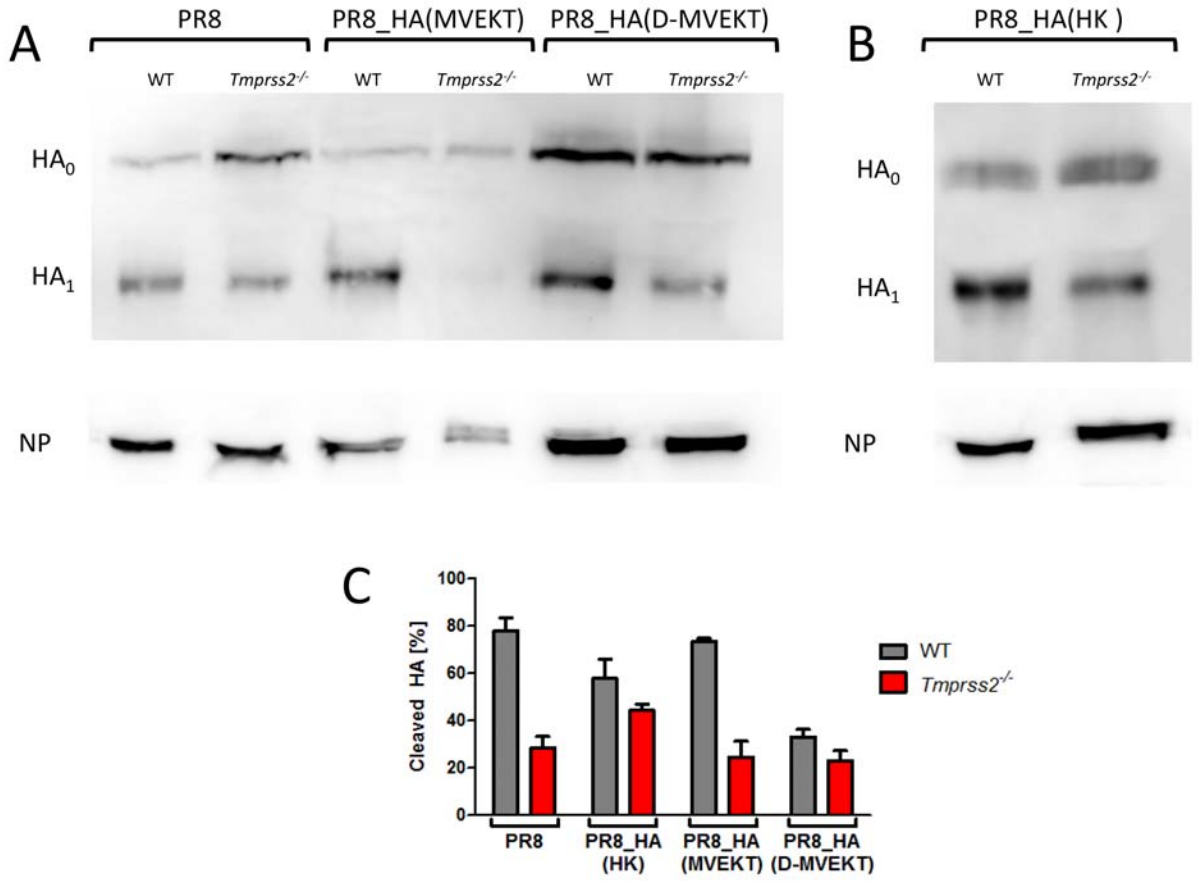

FIG S3: Cleavage of HA from PR8_HA(MVEKT) and PR8_HA(D-MVEKT) viruses in bronco-alveolar lavages (BAL) of infected *Tmprss2*$^{-/-}$ mice.

Female 8-12 week-old WT and *Tmprss2*$^{-/-}$ mice were infected intranasally with $2x10^5$ffu PR8, PR8_HA(MVEKT), and PR8_HA(D-MVEKT), and PR8_HA(HK). On day 3 p.i., BAL samples were prepared and total protein was quantified. For each sample, 20µg total protein were run on a SDS-PAGE, blotted to a PVDF membrane and stained by (A) anti-H1N1 antibody (PR8), (B) anti-H3N2 antibody (A/Brisbane/10/2007) or anti-NP antibody (corresponding NP bands are shown below each sample in A and B). HA_0: uncleaved HA, HA_1: N-terminal part of cleaved HA. Detection of signals was performed using the FujiFilm LAS-3000 imaging system. (C) Cleaved HA is shown as the ratio of HA1*100/(HA1+HA0). Band intensities of four blots derived from the same BAL sample were analyzed using ImageJ software and the mean was depicted ± 1 SEM.

Mutagenesis primer

HA modification	Primer name	Sequence in 5'->3' orientation	template plasmid	resulting plasmid
EKT	Mut EKT F	5‘-GGTTACAGGACTAAGGAACATTCCGGA GAAGCAAACCAGAGGTCTATTTGGAGCC-3‘	pHW-HA	pHW-HA(EKT)
	Mut EKT R	5‘-GGCTCCAAATAGACCTCTGGTTTGCTT CTCCGGAATGTTCCTTAGTCCTGTAACC-3‘		
MVEKT	Mut MVEKT F	5'-CCAAATTGAGGATGGTTACAGGAaTgA GGAACgTTCCGgagAagCAAaCCAGAGG-3'	pHW-HA(EKT)	pHW-HA(MVEKT)
	Mut MVEKT R	5'-CCTCTGGtTTGctTctcCGGAAcGTTCCT cAtTCCTGTAACCATCCTCAATTTGG-3'		
DMVEKT	Mut D F	5'-CACTGTTGACACAGTACTCGAtAAGAAT GTGACAGTGACACAC-3'	pHW-HA(MVEKT)	pHW-HA(DMVEKT)
	Mut D R	5'-GTGTGTCACTGTCACATTCTTaTCGAGT ACTGTGTCAACAGTG-3'		
D	Mut D F	5'-CACTGTTGACACAGTACTCGAtAAGAAT GTGACAGTGACACAC-3'	pHW-HA	pHW_HA(D)
	Mut D R	5'-GTGTGTCACTGTCACATTCTTaTCGAGT ACTGTGTCAACAGTG-3'		

Cloning primers

Segment	Name	Sequence in 5'->3' orientation	annealing temperature	elongation time
1	SLIC PB2 F	5'-gacctccgaagttgggggggAGCGAAAGCAGGTCAAWTATATTCA-3'	58	4 min
	SLIC20+13R	5'-ttttgggccgccgggttattAGTAGAAACAAGG-3'	58	4 min
2	SLIC PB1 F	5'-gacctccgaagttgggggggAGCGAAAGCAGGCAAACCATTTGATG-3'	58	1 min
	SLIC PB1 R	5'-ttttgggccgccgggttattAGTAGAAACAAGGCATTTTTTCAYG-3'	58	1 min
3	SLIC PA F	5'-gacctccgaagttgggggggAGCRAAAGCAGGTACTGATYCRAATG-3'	58	1 min
	SLIC PA R	5'-ttttgggccgccgggttattAGTAGAAACAAGGTACTTTTTTGGACA-3'	58	1 min
4	SLIC HA F	5'-ggtcgacctccgaagttgggggggAGCAAAAGCAGGGG-3'	60	1.5 min
	SLIC HA R	5'-ggcattttgggccgccgggttattAGTAGAAACAAGGGTGTTTT-3'	60	1.5 min
5	SLIC20+12F	5'-gacctccgaagttgggggggAGCAAAAGCAGG-3'	57	1 min
	SLIC20+13R	5'-ttttgggccgccgggttattAGTAGAAACAAGG-3'	57	1 min
6	SLIC20+12F	5'-gacctccgaagttgggggggAGCAAAAGCAGG-3'	58	1 min
	SLIC20+13R	5'-ttttgggccgccgggttattAGTAGAAACAAGG-3'	58	1 min
7	SLIC20+12F	5'-gacctccgaagttgggggggAGCAAAAGCAGG-3'	58	1 min
	SLIC20+13R	5'-ttttgggccgccgggttattAGTAGAAACAAGG-3'	58	1 min
8	SLIC20+12F	5'-gacctccgaagttgggggggAGCAAAAGCAGG-3'	57	1 min
	SLIC20+13R	5'-ttttgggccgccgggttattAGTAGAAACAAGG-3'	57	1 min
